# A case of multiple hepatic angiomyolipomas with high ^18^ F-fluorodeoxyglucose uptake

**DOI:** 10.1186/1471-2342-14-17

**Published:** 2014-05-20

**Authors:** Soma Kumasaka, Yukiko Arisaka, Azusa Tokue, Tetsuya Higuchi, Takahito Nakajima, Yoshito Tsushima

**Affiliations:** 1 Department of Diagnostic Radiology and Nuclear Medicine, Gunma University Graduate School of Medicine, Showa-machi, Maebashi 3-39-22 Gunma, Japan

**Keywords:** Hepatic angiomyolipoma, FDG, PET, HMB-45

## Abstract

**Background:**

Hepatic angiomyolipoma is a rare benign mesenchymal tumor. We report an unusual case of a patient with multiple hepatic angiomyolipomas exhibiting high ^18^ F-fluorodeoxyglucose (FDG) uptake.

**Case presentation:**

A 29-year-old man with a medical history of tuberous sclerosis was admitted to our hospital for fever, vomiting, and weight loss. Abdominal dynamic computed tomography revealed faint hypervascular hepatic tumors in segments 5 (67 mm) and 6 (10 mm), with rapid washout and clear borders; however, the tumors exhibited no definite fatty density. Abdominal magnetic resonance imaging revealed that the hepatic lesions were slightly hypointense on T1-weighted imaging, slightly hyperintense on T2-weighted imaging, and hyperintense with no apparent fat component on diffusion-weighted imaging. FDG-positron emission tomography (PET) imaging revealed high maximum standardized uptake values (SUVmax) of 6.27 (Segment 5) and 3.22 (Segment 6) in the hepatic tumors. A right hepatic lobectomy was performed, and part of the middle hepatic vein was also excised. Histological examination revealed that these tumors were characterized by the background infiltration of numerous inflammatory cells, including spindle-shaped cells, and a resemblance to an inflammatory pseudotumor. Immunohistochemical evaluation revealed that the tumor stained positively for human melanoma black-45. The tumor was therefore considered an inflammatory pseudotumor-like angiomyolipoma. Although several case reports of hepatic angiomyolipoma have been described or reviewed in the literature, only 3 have exhibited high ^18^ F-FDG uptake on PET imaging with SUVmax ranging from 3.3–4.0. In this case, increased ^18^ F-FDG uptake is more likely to appear, particularly if the inflammation is predominant.

**Conclusion:**

Although literature regarding the role of ^18^ F-FDG-PET in hepatic angiomyolipoma diagnosis is limited and the diagnostic value of ^18^ F-FDG-PET has not yet been clearly defined, the possibility that hepatic angiomyolipoma might exhibit high ^18^ F-FDG uptake should be considered.

## Background

Angiomyolipoma (AML) of the liver is a rare benign mesenchymal tumor with positive human melanoma black-45 (HMB-45) expression and a heterogeneous composition of blood vessels, and smooth muscle cells, and adipose tissue cells, which account for the tumors varying morphological features [1]. Most AMLs contain various amount of fat that enable detection via computed tomography (CT) or magnetic resonance imaging (MRI) [2-5]. However, some hepatic AMLs have low fat contents, posing difficulties for establishing a definitive preoperative diagnosis [6,7]. Diagnostic functional positron emission tomography (PET) imaging using an ^18^ F-fluorodeoxyglucose (FDG) tracer is a useful technique for detecting and differentiating benign and malignant tumors. Similar to benign tumors, which tend to exhibit low FDG uptake, hepatic AMLs have been reported to exhibit similarly low uptake levels in the absence of hemorrhage [8]. However, we encountered a distinct case with multiple AMLs with high FDG uptake in the liver of a 29-year-old man.

## Case presentation

A 29-year-old man with a medical history of tuberous sclerosis was admitted to our hospital for fever, vomiting, and weight loss (4 kg/month). His serum C-reactive protein and hepatic enzyme levels were increased. Tests for all evaluated markers of hepatic viruses and tumors such as alpha-fetoprotein, carcinoembryonic antigen, protein induced by vitamin K absence-2, and cancer antigen 19–9 were negative.

**Figure 1:**
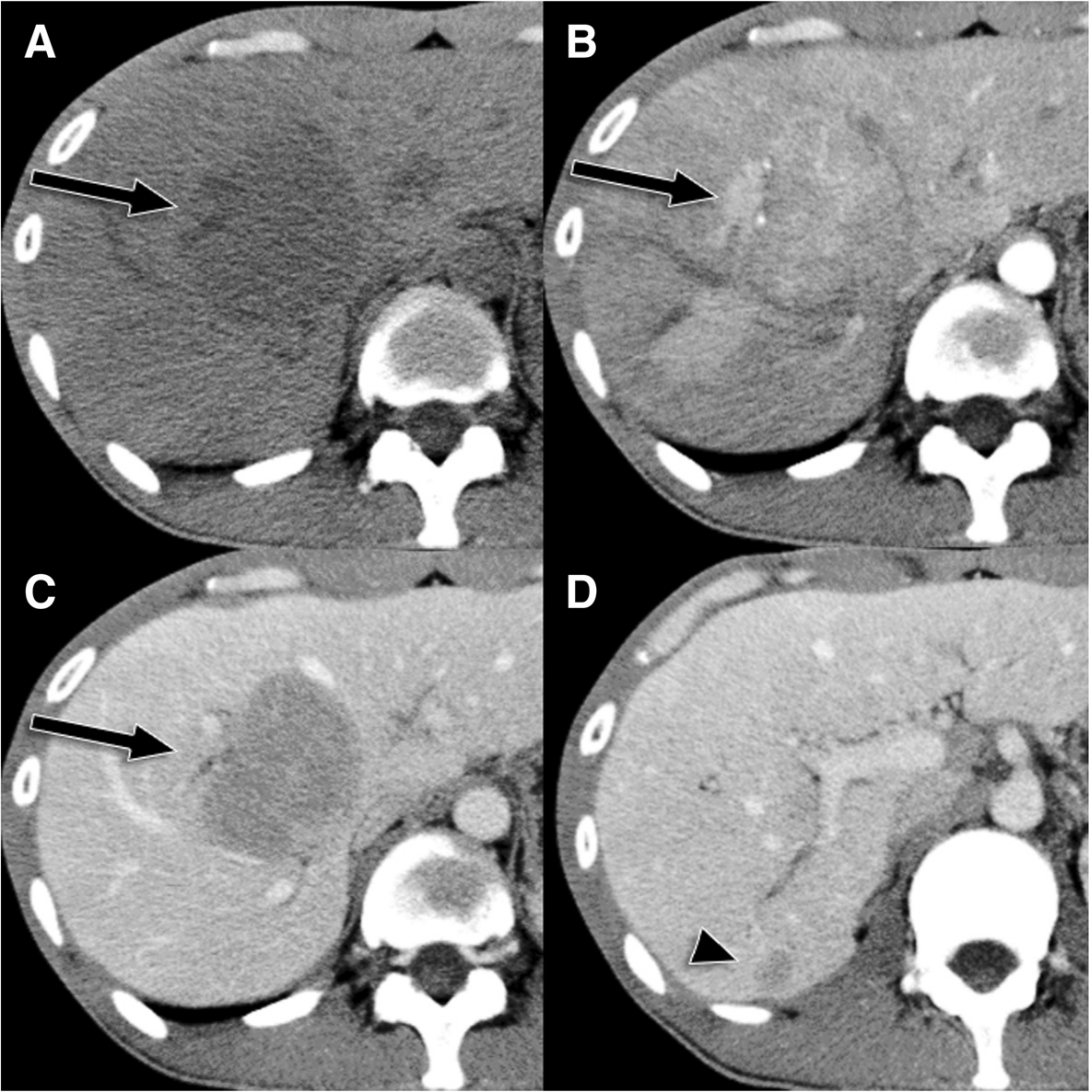
**Abdominal computed tomography. (A)** Axial computed tomography (CT) imaging shows a liver lesion (arrow) with low attenuation. **(B)** On early-phase axial contrast-enhanced CT imaging, the lesion exhibits heterogeneous enhancement. **(C)** On portal-phase axial contrast-enhanced CT imaging, the lesion shows rapid washout in segment 5. **(D)** The segment 6 lesion exhibits a similar pattern (arrowhead).

Abdominal dynamic CT revealed faint hypervascular hepatic tumors in segments 5 (S5; 67 mm) and 6 (S6; 10 mm), with rapid washout and clear borders but without definite fatty densities. CT also revealed multiple tumors with fat components in both kidneys, but these did not show definite enhancements (Figure 1).

**Figure 2:**
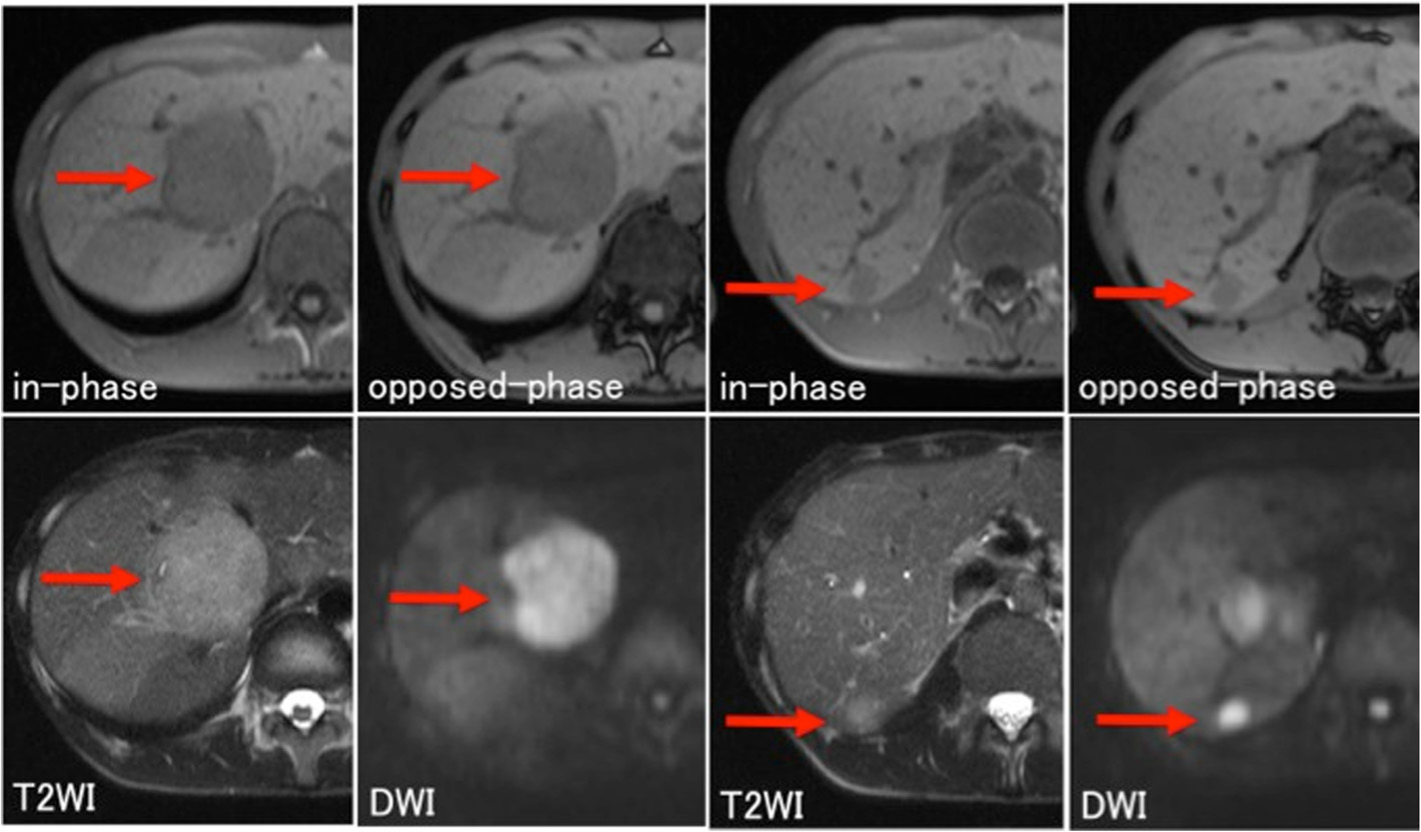
**Abdominal magnetic resonance (MR) imaging.** Abdominal magnetic resonance (MR) imaging revealed that the hepatic tumors (segment 5: arrow, segment 6: arrowhead) were slightly hypointense on T1-weighted imaging (WI), slightly hyperintense on T2WI, and hyperintense without an apparent fat component on diffusion-weighted imaging.

^18^ F-FDG PET/CT (Biograph 16; Siemens Medical Solutions, Erlangen, Germany) imaging revealed that the hepatic tumors exhibited high maximum standard uptake values (SUVmax) of 6.27 (S5) and 3.22 (S6; Figure 3). No definite FDG-uptake was observed in the renal AMLs.

Abdominal MR imaging (1.5 T Magnetom Symphony; Siemens Medical Solutions, Erlangen, Germany) revealed that the hepatic lesions were slightly hypointense on T1-weighted imaging (T1WI), slightly hyperintense on T2-weighted imaging (T2WI), and hyperintense on diffusion-weighted imaging (DWI) with no apparent fat component (Figure 2). The tumors in both kidneys were mildly hypointense on both T1WI and T2WI and mildly hyperintense with fat components on DWI.

**Figure 3:**
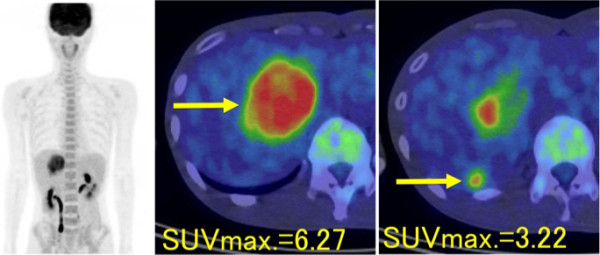
^**18**^**F-fluorodeoxyglucose (FDG) positron emission tomography (PET)/computed tomography (CT) imaging of hepatic angiomyolipomas.** The fused PET/CT image demonstrates the markedly increased FDG uptake in segments 5 and 6 of the liver.

Although a preoperative percutaneous biopsy was performed and a histological examination of the specimens suggested the diagnosis of angiomyolipoma, the high FDG uptake and increasing tumor sizes made it difficult to exclude a malignant transformation of angiomyolipoma. A right hepatic lobectomy was performed and a part of the middle hepatic vein was also excised. The patient recovered well after surgery. His initial symptoms resolved completely, and his serological parameters of inflammation returned to normal by the time of release from hospital. Currently, the patient remains in a healthy condition and has exhibited no signs of recurrence at 4 years after surgery.

**Figure 4:**
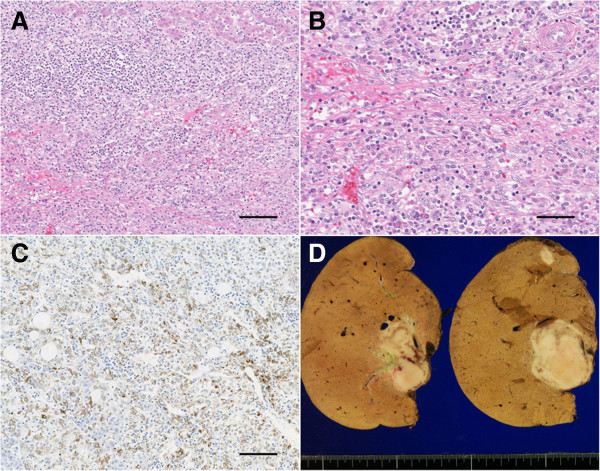
**Histological features of hepatic angiomyolipomas. (A, B)** Histological examination revealed the background infiltration of numerous inflammatory cells, including spindle-shaped cells (A: original magnification = 20×, scale bar = 50 μm, B: 40×, scale bar = 100 μm; hematoxylin–eosin staining). **(C)** The immunohistochemical analysis revealed positive staining for human melanoma black-45 (HMB-45; original magnification = 20×, scale bar = 50 μm). **(D)** In a gross examination, the cut AML surfaces revealed well-delineated borders and slightly variegated appearances.

Histological examination revealed that these tumors were characterized by the background infiltration of numerous inflammatory cells, including spindle-shaped cells, and an absence of fat (Figure 4) and thus resembled inflammatory pseudotumors. On immunohistochemical analysis, the tumors stained positively for HMB-45, CD31, vimentin, and α-smooth muscle actin. However, the tumor cells were negative for keratin, epithelial membrane antigen, hepatocyte paraffin1, c-kit, CD34, CD56, and S-100. The tumor was hence diagnosed as an inflammatory pseudotumor-like AML.

## Discussion

AML is a benign mesenchymal tumor that has reportedly occurred frequently in the kidney but rarely in the liver. Approximately 5%–10% of hepatic AML are associated with renal AML and tuberous sclerosis (Bourneville’s disease), and this was true for our patient. The first case of hepatic AML was described by Ishak in 1976 [9]. Although fatty tissue is a characteristic of AML, the fat content within the tumor can range from 5% to 90% of the tumor volume, thus resulting in varying radiological appearances [2,5,6,10]. Preoperative diagnosis relies on imaging studies, which include CT and MRI. However, it is often difficult to distinguish these lesions from other hepatic fatty tumors such as fat-containing hepatic adenomas or hepatocellular carcinomas with fatty metamorphosis.

Although AMLs are considered benign, malignant transformation is not a rare complication associated with renal AML [11]. In recent years, the malignant transformation of hepatic AML has also been reported in the literature [12,13]. ^18^ F-FDG PET/CT is useful for detecting malignant transformation [14]. Moreover, because hepatic AML has been reported to exhibit low ^18^ F-FDG-uptake [8], ^18^ F-FDG PET/CT might be highly valuable for excluding malignancies among hepatic lesions suspected to be AML.

We performed a Medline search using the search terms “liver”, “angiomyolipoma”, and “FDG”. Although several case reports of hepatic AML have been described or reviewed in the literature, only 3 cases exhibited high ^18^ F-FDG uptake on PET imaging, with SUVmax ranging from 3.3–4.0 [8,13,15-20] (Table 1).

**Table 1:** Reports of FDG uptake in hepatic AMLs to the present date

**Author**	**Age**	**Gender**	**Tumor size (cm)**	**SUVmax**
Takanami [8]	74	Female	20	Low
	62	Female	15	4.0
Awane [15]	48	Female	6	Low
Kubo [16]	70	Female	3.2	1.9
Kinugasa [17]	42	Female	1.4	3.9
Sakaguchi [18]	80	Female	4.3	3.3
Kawaoka [19]	41	Female	3.9	1.9
Lhommel R [20]	64	-	7	Low
Lee JH [6]	47	Female	4	Low

FDG, ^18^ F-fluorodeoxyglucose; AML, angiomyolipoma; SUVmax, maximum standard uptake value.

Kinugasa *et al*. [17] suggested that glucose hypermetabolism in smooth muscle cells and a high cell density due to the lack of a fat component might increase the ^18^ F-FDG uptake. Takanami *et al.*[8] suggested that hepatic AMLs might exhibit increased ^18^ F-FDG uptake in the presence of hemorrhage or an inflammatory response. In such cases, increased ^18^ F-FDG uptake is more likely to appear, particularly if the inflammatory tissue is predominant. Because the average mean SUV of the normal liver is approximately 3.5 ± 3.1 and the malignant lesion-to-liver SUV ratio is approximately 1.9 ± 1.4 [21], the FDG uptakes of the angiolipomas described in Table 1 were easily obscured by the FDG uptake of the normal liver tissue. In our case, the angiolipomas exhibited apparently high levels of FDG uptake.

Recently, some hepatic epithelioid AMLs have been reported to have features similar to those of renal AMLs reported previously. Epithelioid AMLs either lack or contain only a minimal amount of adipose tissue and are thus more difficult to distinguish from other hypervascular tumors. The imaging features of epithelioid AMLs include an absent capsule and hypervascularity with central punctiform or filiform vessels that exhibit characteristic enhancement [22,23]. No literature reports have described the FDG-PET imaging features of epithelioid AMLs. In our case, although the CT and MRI imaging features were similar to those of epithelioid AML, no epithelial cells were observed in a histopathological examination.

The presence of HMB-45-positive smooth muscle cells in an immunostaining analysis is the definitive criterion for diagnosing hepatic AML [24]. Although AML was already suggested by the findings the preoperative percutaneous biopsy, we could not completely rule out the possibility of malignancy because of the atypical MRI findings and high ^18^ F-FDG uptake in the lesions.

## Conclusion

We report herein a case of multiple hepatic angiomyolipomas with high ^18^ F-FDG uptake. Although the literature reports on the role of ^18^ F-FDG PET/CT in hepatic AML diagnosis are limited and the diagnostic value of ^18^ F-FDG PET/CT has not yet been clearly defined, the possibility that hepatic AML might exhibit high ^18^ F-FDG uptake should be considered.

## Consent

Written informed consent for the publication of this case report and any accompanying images was obtained from the patient. A copy of this written consent is available for review by this journal.

## Abbreviations

PET: Positron emission tomography; CT: Computed tomography; FDG: Fluorodeoxyglucose; MRI: Magnetic resonance imaging; SUV: Standardized uptake values; SUVmax: Maximum standardized uptake values; T1WI: T1-weighted imaging; T2WI: T2-weighted imaging; DWI: Diffusion-weighted imaging; AML: Angiomyolipoma; HMB-45: Human melanoma black-45.

## Competing interests

The authors declare that they have no competing interests.

## Authors’ contributions

SK, YA, and TN drafted the manuscript. SK, YA, AT, and TH contributed to the diagnosis. YA and TN reviewed the radiological findings and interpreted the data. YA conceived the study. YA, TN, and YT reviewed the manuscript. All authors approved the final version of the manuscript.

## Pre-publication history

The pre-publication history for this paper can be accessed here:

http://www.biomedcentral.com/1471-2342/14/17/prepub
